# Levels of Bisphenol A and its analogs in nails, saliva, and urine of children: a case control study

**DOI:** 10.3389/fnut.2023.1226820

**Published:** 2023-08-14

**Authors:** Yolanda Gálvez-Ontiveros, Inmaculada Moscoso-Ruiz, Vega Almazán Fernández de Bobadilla, Celia Monteagudo, Rafael Giménez-Martínez, Lourdes Rodrigo, Alberto Zafra-Gómez, Ana Rivas

**Affiliations:** ^1^Department of Nutrition and Food Science, University of Granada, Granada, Spain; ^2^Instituto de Investigación Biosanitaria ibs. GRANADA, Granada, Spain; ^3^"José Mataix Verdú" Institute of Nutrition and Food Technology (INYTA), Biomedical Research Centre (CIBM), University of Granada, Granada, Spain; ^4^Department of Analytical Chemistry, University of Granada, Granada, Spain; ^5^Heath Center of Maracena, Granada, Spain; ^6^Department of Legal Medicine and Toxicology, University of Granada, Granada, Spain

**Keywords:** overweight and obesity, childhood obesity, obesogens, BPA and analogs, biological samples

## Abstract

**Introduction:**

A growing number of studies link the increase in overweight/obesity worldwide to exposure to certain environmental chemical pollutants that display obesogenic activity (obesogens). Since exposure to obesogens during the first stages of life has been shown to have a more intense and pronounced effect at lower doses, it is imperative to study their possible effects in childhood. The objective here was to study the association of Bisphenol A (BPA) and 11 BPA analogs in children, using three biological matrices (nails, saliva and urine), and overweight and obesity (*n* = 160).

**Methods:**

In this case–control study, 59 overweight/obese children and 101 controls were included. The measuring of Bisphenols in the matrices was carried out by ultra-high performance liquid chromatography coupled with triple quadrupole tandem mass spectrometry (UHPLC-MS/MS). Logistic regression was used to study the association between overweight/obesity and Bisphenol exposure.

**Results:**

The results suggested that BPF in nails is associated with overweight/ obesity in children (OR:4.87; *p* = 0.020). In saliva, however, the highest detected concentrations of BPAF presented an inverse association (OR: 0.06; *p* = 0.010) with overweight/obesity. No associations of statistical significance were detected between exposure to BPA or its other analogs and overweight/obesity in any of the biological matrices.

## 1. Introduction

Overweight and obesity are defined by the World Health Organization (WHO) as “an abnormal or excessive accumulation of fat that may be harmful to health.” The prevalence of overweight and obesity has tripled in most of the world’s population since 1975 (1). Therefore, according to the WHO, obesity is one of the most important public health problems in the world today (2). Obesity is known to be a major risk factor in the development of cardiovascular disease, several types of cancer, diabetes and premature death, among other associated problems (1, 3, 4).

All the triggers and mechanisms involved in the development of obesity are not yet fully understood (5). The main cause of the onset of obesity is an imbalance between consumed and expended calories (1). Obesity has also been linked to genetic factors (6, 7). However, in these last few decades evidence has mounted linking the increase of obesity worldwide to exposure to obesogens (8, 9). Obesogens are understood to be environmental chemicals that promote inadequate fat storage through their interference with adipogenesis, as well as interfering with mechanisms controlling satiety, appetite and food preferences, among others (10–13). Bisphenol A (BPA) is among the most studied obesogens.

Bisphenols are produced in large quantities worldwide (>5 million tons per year) and their use has been increasing in the last decades. Due to this ubiquity, bisphenols have been detected in food, dust, sludge, drinking water, etc. “(14)” and the main route of human exposure to them is diet (15, 16). Bisphenols are constituents of polycarbonate plastics and epoxy resins, used to make varnishes, lacquers, adhesives, plastics, dental sealants, water pipes and food packaging. However, their presence in the latter is not stable and over time can migrate from the packaging to the food (14, 17). In 2011, the European Commission banned the use of BPA in the manufacture of polycarbonate infant feeding bottles (18). This is because the early stages of development and childhood are the most vulnerable to exposure to environmental chemical contaminants, as it has been shown that the effect is more intense and pronounced in children at lower doses. This is largely because certain protective mechanisms present in adulthood, such as detoxifying liver enzymes and the blood–brain barrier, are not fully developed in the fetal and postnatal stage. Additionally, metabolism is higher in these early stages of development than in the later stages, enhancing the effects of these environmental chemical pollutants (19). In 2015, the European Food Safety Authority (EFSA) reduced the tolerable daily intake (TDI) of BPA from 50 to 4 micrograms per kilogram of body weight per day. In 2018, the EU Regulation on the use of BPAs in varnishes and coatings in contact with food was adopted, prohibiting those specifically intended to come into contact with food for infants. Additionally, a tolerable limit of 0.05 mg BPA per kilogram of food was set for plastic materials in contact with food, and further guidelines were established to ensure that exposure to BPAs remains below the TDI (20). Currently, EFSA has re-evaluated the risks of BPA and has lowered the TDI from 4 micrograms per kilogram of body weight per day to 0.2 nanograms per kilogram of body weight. As a result of the alarm generated by the adverse effects associated with BPA exposure, BPAs have begun to be replaced by their analogs. Information on the toxic potential of these analogs is still very deficient, though since they have a very similar chemical structure to BPAs, they are expected to exhibit similar endocrine disrupting and obesogenic activity as BPA, as several studies have shown (12, 21–24).

*In vitro* studies have shown that BPA and analogs [Bisphenol S (BPS) and Bisphenol F (BPF)] promote adipocyte differentiation, leading to excessive fat accumulation. This effect was observed from concentrations of 10 nM to 50 μM (22, 23, 25). Animal studies, at concentrations of 2.4 and 25 μg BPA per kilogram of body weight, have found that BPA exposure increases adipose tissue mass and promotes weight gain (26, 27). Numerous epidemiological studies have focused on BPA as an obesogen, showing that BPA could exert effects on all organs involved in the regulation of energy homeostasis, such as adipose tissue and the brain, among others (8). In these studies, exposure to low-dose BPA and its analogs was associated with weight gain, disruption of carbohydrate and lipid homeostasis, as well as having an effect on brain regions involved in food intake (8, 22, 23).

Few epidemiological studies focused on the effects of BPA and its analogs on obesity/overweight in children, and all of said studies were restricted to urine as the biological matrix. The focus of this research was thus to study the association between the presence of BPA and its analogs and obesity/overweight in children, using three distinct biological matrices (nails, saliva, and urine).

## 2. Materials and methods

### 2.1. Study design and setting

This case–control study was designed to evaluate environmental factors affecting the overweight and obesity in Spanish children and adolescents, funded by ‘FEDER-Consejería de Salud y Familias’ of the Junta de Andalucía PE-0250-2019. Recruitment of the study population was carried out between January 2020 and January 2022 in various health and educational centers in the province of Granada, Spain. The parents or legal guardians of the participants gave their written informed consent. Confidentiality was guaranteed with the deletion of participants personal data. The study was approved by the Ethics Committee of the University of Granada.

### 2.2. The study population

Eligible cases met the following inclusion criteria: overweight or obesity diagnosis; between the ages of 6 and 12 years-old; having resided continuously in the study areas for at least 6 months. The same inclusion criteria was applied to the controls, with the exception of a diagnosis of overweight or obesity. Exclusion criteria included obesity as a symptom of other pathologies, or as a side effect of pharmacological treatment.

Of the 231 who agreed to participate, the selected subjects were those that correctly collected and submitted biological samples (saliva, urine and nails), amounting to 160 participants (53.5% male). After comparing total population with selected sample (subjects with the three biological samples correctly collected), non-significant differences were observed for gender, age, weight, height and urinary creatinine level, both for cases and for controls groups (Supplementary Table S1).

### 2.3. Data collection

The variables taken into account in the study were anthropometry (weight and height), sociodemographic variables (gender and age), urine creatinine levels and levels of Bisphenols in biological matrices.

Anthropometric measurements were taken by qualified personnel. Height was taken with a measuring rod [model SECA 214 (20–207 cm)], while weight was measured with a portable Tanita scale (model MC 780-S MA). Body mass index (BMI) was calculated as weight in kg divided by height squared, in meters. Participants were classified as underweight, normal weight, overweight and obese using the standards proposed by the International Obesity Task Force, as described by Cole et al. (28, 29).

The determination of creatinine levels in the urine samples was analyzed by The Ángel Méndez Soto Clinical Analysis Laboratory. The method used was the classical Jaffé method, based on photometric measurement of the reaction kinetics of creatinine with picric acid at 37°C (30, 31). Biosystems provided a reagent kit (Barcelona, Spain).

### 2.4. Determination of bisphenols in biological samples

The biological samples used were saliva, urine and nails. For saliva collection, each subject was given a wide-mouth glass bottle, and for the duration of a week, they had to passively collect saliva on an empty stomach until the bottle was approximately half-full. Urine was collected in a polypropylene bottle. A single urine sample is taken from the study subjects, this should be the first urine of the day. Saliva and urine were stored in the participants’ homes under frozen conditions until collection. For the nail samples the participants were given a bottle to collect both finger and toenails, over a 3-month period. The nails are collected without nail polish. After collection, all samples were stored at −80°C until their laboratory analysis, with the exception of the nails that were kept at room temperature.

A total of 12 Bisphenols (BPA; BPF; BPS; Bisphenol AP, BPAP; Bisphenol AF, BPAF; Bisphenol B, BPB; Bisphenol E, BPE; Bisphenol C, BPC; Bisphenol FL, BPFL; Bisphenol M, BPM; Bisphenol P, BPP; Bisphenol Z, BPZ) were analyzed for.

Following the completion of the questionnaire, all samples were collected within a 1 to 4 month-period. The validation parameters, LOQ, LOD, calibration range, recovery, etc., can be corroborated in our research group’s previously published studies (32–34).

#### 2.4.1. Determination of bisphenols in saliva

The method followed for the determination of Bisphenols in saliva (*n* = 89) was developed by members of the research group (33). 1 g of saliva was deposited in a 10 mL glass tube. Subsequently, 2 mL of acetonitrile and 150 μL of acetic acid solution (0.1 M) were added. This mixture was vortexed and centrifuged for 5 min. The supernatant was recovered and transferred to a 10 mL glass tube, then evaporated to dryness. Subsequently, the first extraction was carried out by adding 1.5 mL of acetone extraction solvent to the dry residue. Then ultrasound-assisted extraction was carried out for 30 min at a power setting of 35%. The mixture was then centrifuged for 5 min, and the supernatant was recovered by transferring it to another glass tube. Extraction was performed a second time, using 1.5 mL ethanol as the extraction solvent under the same conditions. The supernatant was evaporated to achieve complete dryness, then it was reconstituted with 20 μL of methanol (MeOH) and 80 μL of ultrapure water. Finally it was centrifuged and analyzed using ultra-high performance liquid chromatography coupled with a triple quadrupole tandem mass spectrometry (UHPLC-MS/MS) system (31).

#### 2.4.2. Determination of bisphenols in urine

The urine samples were submitted previously to an enzymatic treatment (β-glucuronidase and β-glucuronidase/arylsulfatase), the enzymatic treatment followed is described in the work of Moscoso-Ruiz et al. (34) and Vela-Soria et al. (35).

For the determination of Bisphenols in the urine (n = 149) we used the improved optimization of the extraction method for endocrine disrupting chemicals (EDCs) as described in Vela-Soria et al. (35) and further developed as per Moscoso-Ruiz et al. (34). 4 mL of 10% (w/v) NaCl and 100 μL of HCl (6 N) were added to 4 mL of urine until pH 2 was reached. Subsequently, dispersive microliquid-liquid extraction was performed, with the addition of a mixture of 600 μL of chloroform and 400 μL of acetone, injected directly into the urine sample using a Hamilton syringe. The mixture was then vortexed gently and centrifuged for 5 min. The sedimented phase was recovered, then transferred to another 10 mL glass tube. This extraction process was repeated three times, and the resulting organic phase was then evaporated to dryness (sedimented phase). The dried residue was reconstituted with 20 μL of MeOH and 80 μL of ultrapure water, centrifuged and analyzed on a UHPLC-MS/MS system (34).

#### 2.4.3. Determination of bisphenols in nails

The method for the determination of Bisphenols in nails (*n* = 74) was also developed by members of this research group as described in the article Martín-Pozo et al. (32). Firstly, the nails were washed following the protocol described in the work of Martin-Pozo et al. (32) so that any external contamination was removed. Then 0.1 g of lyophilized and shredded nails were weighed and 1 mL of sodium hydroxide/MeOH (0.04 mol L^−1^) was added, shaken in a vortex for 2 min and incubated at 30°C for 15 h. After incubation, the digested nails were cooled to room temperature. Subsequently, they were centrifuged for 10 min, the organic phase was then recovered and evaporated to dryness. The residue was reconstituted, using 20 μL of MeOH and 80 μL of ultrapure water, then it was centrifuged and analyzed on a UHPLC-MS/MS system (32).

### 2.5. Statistical analysis

The distribution of the continuous and parametric variables (height) was summarized via mean and standard deviation (SD), while the distribution of the continuous and non-parametric variables (weight, urinary creatinine levels and Bisphenol concentrations in the biological samples) was summarized via the median and interquartile range (IQR). Frequency distributions were calculated for the categorical variables (sex and age).

To evaluate the differences between cases and controls for all variables, the Student’s *t*-test (for parametric variables), Mann–Whitney *U*-test (for non-parametric variables) and Pearson’s Chi-square test (for categorical variables) were used.

A logistic regression model was used to analyze the influence of Bisphenol concentrations (ng g^−1^ or ng mL^−1^) in the three biological matrices as the independent variable, and on overweight and obesity as the dependent variable. The independent variables were dichotomized according to the median value (reference category: concentration ≤ median value). When the % of undetected concentrations of an analyte was >30%, the cut-off point for the dichotomisation was the limit of detection (LOD)/√2 (reference category: concentration ≤ LOD/√2) (36). LOD value for total Bisphenol in urine was considered as the sum of each Bisphenol LOD separately.

Odds ratios (OR) and standard error (S.E.) were calculated for the initial and adjusted models. Gender, age (for nails, urine and saliva) and creatinine levels (for urine analysis) were considered confusion factors (CF) in the adjusted models.

SPSS v.23 (version 23, IBM® SPSS® Statistics, Armonk, NY, United States) was employed for all the statistical analyses. The significance was set to *p* < 0.05.

## 3. Results

Table 1 displays the study population’s characteristics. Significant differences were observed for anthropometric variables, the values being higher for cases than for controls (*p* < 0.001) for all three parameters (weight, height, and BMI). The population distribution according to gender and age, and median values for urinary creatinine levels, did not show significant differences between cases and controls.

**Table 1:** General characteristics of study population (*N* = 160).

		*n*	Controls (*n* = 101)	Cases (*n* = 59)	*p*
Gender (%)	Male	85	58.80	41.20	0.505^b^
	Female	76	67.10	32.90	
Age, categorized (%)	≤10 years	125	64.00	36.00	0.280^b^
	>10 years	36	58.30	41.70	
Weight, kg	Median		25.45	53.30	**<0.001**^**c**^
	IQR		12.58	21.90	
Height, cm	Mean		127.79	140.37	**<0.001**^**a**^
	SD		20.68	12.93	
BMI, kg/m^2^	Mean		16.14	24.45	**<0.001**^**a**^
	SD		2.03	3.84	
Urinary creatinine, g L^−1^	Median		0.87	0.90	0.439^c^
	IQR		0.60	0.77	

IQR, interquartile range; SD, standard deviation; *p*-values < 0.05 are highlighted in bold; ^a^Student’s *t*-test; ^b^Chi-square test; ^c^*U* Mann–Whitney test.

Table 2 shows the concentrations of Bisphenols determined in the nails, urine and saliva. BPA and BPAF were detected in all three matrices, followed by BPF which was detected in the nails and urine, BPS in the urine and saliva, and BPAP in the nails and saliva. The analogs BPE, BPB, BPC, BPZ, BPM, BPP and BPFL were only detected in the saliva. One of the most important findings of the study was that the nails were the biological matrix with the highest total concentrations of Bisphenol, BPA and BPF (149.22 ng g^−1^, 136.26 ng g^−1^, and 23.06 ng g^−1^). The highest concentration was found for overweight and obese subjects, with significant differences for BPA and total Bisphenols. In the urine, BPA and total Bisphenol were found in higher concentrations in the control group than in the cases, but without significant differences. However, only BPA in the nails showed significant differences (*p* = 0.005). In the saliva the highest detected value of BPA was determined in subjects with a BMI ≥ 25 kg/m^2^. For total Bisphenols determined in the nails and saliva, the highest values were detected in the cases. Nevertheless, significant differences were found only for total Bisphenols (*p* = 0.011) in the nails. In the urine, the highest concentration of total Bisphenols was found in the control group, but no significant differences were observed.

**Table 2:** Bisphenol concentrations in nails, urine, and saliva (ng g^−1^ or ng mL^−1^).

Nails (ng g^−1^)									
	Controls (*n* = 52)				Cases (*n* = 22)				
	% detected	Median	P_25_	P_75_	% detected	Median	P_25_	P_75_	*p*
BPF	92.31	7.78	5.50	17.02	81.82	12.10	8.37	23.06	0.062
BPE	0	<LOD	<LOD	<LOD	4.55	<LOD	<LOD	<LOD	-
BPA	100	21.18	13.17	32.73	100	43.67	18.47	136.26	**0.005**
BPS	0	<LOD	<LOD	<LOD	4.55	<LOD	<LOD	<LOD	-
BPB	0	<LOD	<LOD	<LOD	0	<LOD	<LOD	<LOD	-
BPC	0	<LOD	<LOD	<LOD	0	<LOD	<LOD	<LOD	-
BPZ	0	<LOD	<LOD	<LOD	0	<LOD	<LOD	<LOD	-
BPAP	1.92	<LOD	<LOD	<LOD	9.10	<LOD	<LOD	<LOD	-
BPAF	3.85	<LOD	<LOD	<LOD	0	<LOD	<LOD	<LOD	-
BPM	0	<LOD	<LOD	<LOD	0	<LOD	<LOD	<LOD	-
BPP	0	<LOD	<LOD	<LOD	0	<LOD	<LOD	<LOD	-
BPFL	0	<LOD	<LOD	<LOD	0	<LOD	<LOD	<LOD	-
Bisphenols total	100	36.20	24.42	55.60	100	70.71	33.61	149.22	**0.011**

Urine (ng mL^−1^)									
	Controls (*n* = 97)				Cases (*n* = 52)				
	% detected	Median	P_25_	P_75_	% detected	Median	P_25_	P_75_	*p*
BPF	2.06	<LOD	<LOD	<LOD	3.85	<LOD	<LOD	<LOD	0.522
BPE	0	<LOD	<LOD	<LOD	0	<LOD	<LOD	<LOD	-
BPA	58.76	0.70	<LOD	2.61	71.15	0.58	0.20	1.98	0.969
BPS	9.28	<LOD	<LOD	<LOD	7.69	<LOD	<LOD	<LOD	0.262
BPB	0	<LOD	<LOD	<LOD	0	<LOD	<LOD	<LOD	-
BPC	0	<LOD	<LOD	<LOD	0	<LOD	<LOD	<LOD	-
BPZ	0	<LOD	<LOD	<LOD	0	<LOD	<LOD	<LOD	-
BPAP	0	<LOD	<LOD	<LOD	0	<LOD	<LOD	<LOD	-
BPAF	5.15	<LOD	<LOD	<LOD	7.69	<LOD	<LOD	<LOD	0.882
BPM	0	<LOD	<LOD	<LOD	0	<LOD	<LOD	<LOD	-
BPP	0	<LOD	<LOD	<LOD	0	<LOD	<LOD	<LOD	-
BPFL	0	<LOD	<LOD	<LOD	0	<LOD	<LOD	<LOD	-
Bisphenols total	59.79	1.95	<LOD	4.10	69.23	1.73	<LOD	3.12	0.594

Saliva (ng g^−1^)									
	Controls (*n* = 58)				Cases (*n* = 31)				
	% detected	Median	P_25_	P_75_	% detected	Median	P_25_	P_75_	*p*
BPF	0	<LOD	<LOD	<LOD	0	<LOD	<LOD	<LOD	-
BPE	3.45	<LOD	<LOD	<LOD	6.45	<LOD	<LOD	<LOD	0.650
BPA	34.48	<LOD	<LOD	0.71	41.94	<LOD	<LOD	0.75	0.333
BPS	6.90	<LOD	<LOD	<LOD	19.35	<LOD	<LOD	<LOD	0.499
BPB	1.72	<LOD	<LOD	<LOD	16.13	<LOD	<LOD	<LOD	-
BPC	1.72	<LOD	<LOD	<LOD	9.68	<LOD	<LOD	<LOD	-
BPZ	3.45	<LOD	<LOD	<LOD	3.22	<LOD	<LOD	<LOD	0.755
BPAP	12.07	<LOD	<LOD	<LOD	3.23	<LOD	<LOD	<LOD	0.106
BPAF	93.1	0.24	0.19	0.50	87.10	<LOD	<LOD	0.24	0.073
BPM	39.65	<LOD	<LOD	0.71	51.61	<LOD	<LOD	<LOD	0.913
BPP	27.59	<LOD	<LOD	0.71	16.13	<LOD	<LOD	<LOD	0.174
BPFL	8.62	<LOD	<LOD	<LOD	3.23	<LOD	<LOD	<LOD	0.313
Bisphenols total	100	2.66	2.33	2.97	100	2.68	2.19	3.18	0.455

LOD, limit of detection; *p*-values < 0.050 are highlighted in bold; *U* de Mann–Whitney test.

Tables 3–5 show the influence of BPF, BPA, BPS, BPAF, and the total Bisphenol concentrations determined in the three biological matrices, on overweight and obesity in the study population. Crude and adjusted OR values were significant for BPF in the nails and BPAF in the saliva. Participants with higher BPF concentrations than the median value for nails had a higher likelihood of excess weight (OR = 3.64, *p* = 0.020; OR = 4.87, *p* = 0.012, crude and adjusted values, respectively; Table 3). On the other hand, an inverse association was observed between BPAF and overweight and obesity. Subjects with concentration of BPAF higher than LOD in the saliva had a lower likelihood to be overweight or obese (OR = 0.06, *p* = 0.010; OR = 0.06, *p* = 0.009, crude and adjusted values, respectively; Table 5). A non-significant association was observed between Bisphenol concentrations in urine and overweight/obesity (Table 4).

**Table 3:** Bisphenol levels in nails as influencing factors on overweight/obesity (logistic regression analysis).

	Crude			Adjusted		
	*p*	OR	S. E.	*p*	OR	S. E.
BPF (Ref. BPF concentration ≤ median)	**0.020**	3.64	0.56	**0.012**	4.87	0.63
BPA (Ref. BPA concentration ≤ median)	0.131	2.21	0.52	0.157	2.14	0.54
Bisphenols Total (Ref. Bisphenols total concentration ≤ median)	0.131	2.21	0.52	0.107	2.44	0.55

Ref., reference category; OR, odds ratio; S. E., Standard error; *p*-values < 0.050 are highlighted in bold. All analytes were adjusted for age and gender.

**Table 4:** Bisphenol levels in urine as influencing factors on overweight/obesity (logistic regression analysis).

	Crude			Adjusted		
	*p*	OR	S. E.	*p*	OR	S. E.
BPF (Ref. BPF concentration ≤ LOD)	0.527	0.53	1.02	0.424	0.43	1.04
BPA (Ref. BPA concentration ≤ LOD)	0.653	0.85	0.35	0.551	0.78	0.42
BPS (Ref. BPS concentration ≤ LOD)	0.225	2.38	0.72	0.268	3.76	1.20
BPAF (Ref. BPAF concentration ≤ LOD)	0.886	0.90	0.75	0.616	0.62	0.94
Bisphenols total (Ref. Bisphenols total concentration ≤ LOD)	0.267	0.66	0.38	0.602	0.79	0.46

Ref., reference category; OR, odds ratio; S. E., Standard error; *p*-values < 0.050 are highlighted in bold. All analytes were adjusted for age, gender and urinary creatinine level.

**Table 5:** Bisphenol levels in saliva as influencing factors on overweight/obesity (logistic regression analysis).

	Crude			Adjusted		
	*p*	OR	S. E.	*p*	OR	S. E.
BPA (Ref. BPA concentration ≤ LOD)	0.527	1.34	0.46	0.601	1.28	0.47
BPAF (Ref. BPAF concentration ≤ LOD)	**0.010**	0.06	1.10	**0.009**	0.06	1.10
Bisphenols total (Ref. Bisphenols total concentration ≤ median)	0.823	1.11	0.45	0.884	1.07	0.45

Ref., reference category; OR, odds ratio; S. E., Standard error; *p*-values < 0.050 are highlighted in bold. All analytes were adjusted for age and gender.

## 4. Discussion

The aim of this study was to determine the presence of BPA and its analogs in nails, urine and saliva and to analyze the association between their concentrations and overweight and obesity in children. The findings suggest that a high concentration of BPF in nails is associated with an increased likelihood of overweight/obesity. However, the concentration of BPAF in saliva is inversely related to body weight. The most relevant results obtained in nails for BPF and in saliva for BPAF cannot be compared with previous studies, since the association in these two biological samples has not been studied to date, to the best of our knowledge.

The results of this study show that children who have a higher concentration of BPF, BPA, BPAF and total Bisphenols in urine have a lower likelihood of overweight/obesity, yet without statistically significant results. However, the highest detected concentrations of BPS in urine were associated with a higher likelihood of overweight/obesity, though also without statistically significant results. Previous similar studies show the following findings: Jacobson et al. (37) observed in children and adolescents that urine BPS concentrations were associated with an increased likelihood of obesity (OR = 1.16; 95% confidence intervals (CI): 1.02–1.32); the findings obtained in the work of Liu et al. show that children in the highest quartile of urine BPS concentrations had a 1.36-fold increased risk (95% CI, 0.53–3.51) of obesity compared to children in lower quartiles (38); Gajjar et al. also showed that urinary BPS concentrations were directly associated with higher % body fat in children at 8 years of age (OR = 1.1; 95% CI: −0.6–2.7) (39); as for urinary BPA concentrations, no association was found with obesity in work by Seo et al. (40), Jacobson et al. (37), Xue et al. (41), and Okubo et al. (42). Regarding the concentrations of bisphenols detected in urine of schoolchildren and adolescents in previous studies, it is observed that the concentration range detected for BPA, BPS and BPF is higher than that detected in the present study, being 1.2–1.6 ng mL^−1^ BPA, 0.3–0.4 ng mL^−1^ BPS and 0.2–0.3 ng mL^−1^ BPA (37–39). Whereas for the present study it is 0.58–0.70 ng mL^−1^ BPA and <LOD BPS and BPF.

Conversely, other previous studies found a positive association between high urinary BPA concentrations and overweight/obesity. In the work of Amin et al. a statistically significant direct association between in urine BPA and the risk of obesity was observed, being 12.48 times higher in children found in the third tercile of BPA vs. children in the first and second terciles (95% CI: 3.36–46.39, *p* < 0.001) (5). Liu et al. also found that for children in the highest quartile of Bisphenols (BPA and BPF) in urine, their risk of obesity was 1.74 times higher (95% CI: 0.92–3.31) for BPA and 1.54 times higher for BPF (95% CI, 1.02–2.32) (38). A study in 63 prepubertal children with exogenous obesity found that obese children with metabolic syndrome had significantly higher urinary BPA levels than obese children without metabolic syndrome, and both obese groups had significantly higher urinary BPA levels than the control group (43). Five other studies also found that higher urinary BPA concentrations were directly associated with a greater likelihood of higher BMI in school-aged children (44–48).

Most related Bisphenol studies only focused on urinary BPA and reported that, in childhood, higher BPA concentrations were associated with higher body fat percentages (5, 38, 43–48). However, this work observed that higher detected concentrations of urinary BPA, BPF, BPAF and total Bisphenols show a trend toward a lower BMI (OR: 0.78; OR: 0.43; OR: 0.62; OR: 0.79, respectively), this association was not statistically significant (*p* = 0.551; *p* = 0.424; *p* = 0.616; *p* = 0.602, respectively). Other recent studies on urinary BPA show the following results. Gajjar et al. observed an inverse association between urinary BPA concentrations and body fat % in children 8 years of age (OR = −1.2; 95% CI: −3.4−1.0) (39). Silva et al. observed in children 6 to 10 years of age that higher concentrations of BPA and total Bisphenols in urine were associated with lower BMI (49). In the case of the work carried out by Malik et al., it was observed that the presence of BPA in urine was associated with both obesity and low weight in children, i.e., children who were found to have the highest BPA concentrations in urine (fourth quartile) were associated with both obese and underweight children (50). In relation to the results obtained in saliva in this study, it was observed that the highest BPAF concentrations were associated with lower BMI, the association being statistically significant (OR = 0.06; *p* = 0.009). In the case of nails, BPF showed a direct and statistically significant association (OR = 4.87; *p* = 0.012).

In this study biological samples analyses were used to measure bisphenols exposure. Although multiple sources of bisphenols exposure exist in children, the diet is considered to be one of the main sources of exposure as we have been demonstrated in other studies (51, 52). Food contamination with these chemicals typically occurs during food processing, packaging, transportation, and storage (53).

The use of urine as the only indicator of exposure to Bisphenols may be responsible for the lack of consistency between the associations of Bisphenols and adiposity in the previous studies, given that there is a large within-subject variation in the concentrations of BPA and its analogs in urine due to the short half-life of Bisphenols; even within the same subject there are variations in urinary concentrations from day to day and even over the course of the same day. A key issue is that some BPA and BPA analogs are metabolized very rapidly in the body (21), therefore, spot urine samples are limited in their ability to reflect long-term exposure levels. Urine is commonly used in studies because of the ease and non-invasive nature of its collection in the child population. Nails, however, share both these advantages in addition to being a better biomarker, since they display long-term exposure without fluctuations in concentration levels within the same day, or from 1 day to another. Therefore, the use of nails as a bioindicator is very promising since, by their nature they reflect long-term cumulative exposure, given their relatively slow growth rate (54). In fact, nails have been widely used in forensic analysis and as a bioindicator of the ingestion of drugs and heavy metals (55–57). Moreover, nail samples offer other advantages over other commonly used biological samples such as blood and urine, namely that their collection is simple and non-invasive, and do not require specialized personnel, transport or storage conditions (58). On the other hand, saliva samples are a good alternative to blood samples for assessing human exposure to toxicants because of their non-invasive nature and because they do not require qualified personnel to collect them (59). Another reason why saliva is an adequate alternative to blood is because saliva is secreted by glands surrounded by blood capillaries that allow the passage of toxicants from blood to saliva (60). Therefore, the concentration of toxicants detected in saliva are an accurate reflection of their concentrations in the blood.

Obesity in general is of great concern worldwide. Since increased dietary intake and sedentary lifestyles alone do not explain its increase globally, particular attention is being paid to a wide variety of environmental chemicals that may play an important role. Experimental models and epidemiological evidence suggest that BPA and some of its analogs (BPS and BPF) may act as environmental obesogens (12, 22–24). It is true that various potential mechanisms of action of Bisphenols during adipogenesis have been reported, but there is no common consensus. Some of these mechanisms of action described in the scientific literature is the action of BPA on the modulation of key regulators of adipogenesis [peroxisome proliferator-activated receptor gamma of preadipocyte (PPARγ), CCAAT/enhancer-binding protein Alpha (C/EBPα), dual leucine zipper-bearing kinase (DLK), lipoprotein lipase (LPL)] through interference with receptor signaling (61–63). In one *in vitro* study, it was observed that BPA enters adipose stem cells and interacts with the estrogen receptor (ER), then translocating to the nucleus, where it increases transcription of key adipogenic genes such as DLK, C/EBPα, PPARγ and LPL, which in turn enhances and accelerates the pathway from human adipose stromal/stem cells to mature adipocyte (61). Another adipogenic effect resulting from BPA exposure in 3 T3-L1 cells may also be mediated by increased glucocorticoid receptor and C/EBPδ transcriptional activity (64). Boucher et al. performed an *in vitro* study in which they demonstrated that BPA exposure induced differentiation of primary human preadipocytes through a non-classical ER pathway rather than via glucocorticoid activation (65). The results showed that BPA induced the differentiation of primary preadipocytes through increased expression of adipogenic markers at the mRNA level and increases the expression levels of factors involved in the transcriptional cascade responsible for the differentiation of primary preadipocytes to adipocytes (65). Another mechanism of action that has been studied is the effect of exposure to BPA and its analogs on perturbations in the synthesis and signaling of peripheral serotonin, especially in the intestine, that may contribute to obesity since serotonin also plays an important role in the energy balance of mammals (66). Thus, Barra et al. proposed that BPA and its analogs could increase the intestinal production of peripheral serotonin in the human organism and could contribute to its obesogenic effects (66). The study by Barra et al. (66) is based on additional experiments to support this hypothesis, in one of those studies it was observed that the exposure of mice with genetic or pharmacological inhibition of ryptophan hydroxylase 1 (Tph1) to BPA, evaluated whether the metabolic deficits induced by BPA, such as obesity, depend on the production of peripheral serotonin, since peripheral serotonin in adipose tissue functions as an obesity hormone that reduces energy expenditure and increases lipid accumulation (67).

The importance of bisphenols derives from their ubiquity, as these compounds are present in most commonly used plastic packaging (polycarbonates) and epoxy resins used in the coating of cans in contact with food (14, 17). Exposure to bisphenols is daily. Bisphenols usually enters the bloodstream via the oral route. Its absorption is immediate and with a bioavailability of more than 70% (68). These are conjugated with glucuronic acid and almost entirely eliminated in the urine (69). Bisphenols are also lipophilic, with studies showing that they bioaccumulate in adipose tissue (12). It is true that, if adipose tissue were taken from an obese person, a higher load of these contaminants would be found than in a non-obese person. However, in this study, biological matrices that are not fatty in nature (urine, nails and saliva) have been taken. Therefore, it is not to be expected that in these matrices there will be a greater bioaccumulation behavior in obese people, as they do not have this lipophilic character. Thus, an important question to raise is the relationship between bisphenol exposure and obesity. As mentioned previously, obese people can be expected to have higher concentrations of pollutants in their organism (70). For this reason, statistical models are adjusted for % fat and other predisposing factors (energy intake, physical inactivity, etc.) to obtain the probability of obesity due to bisphenols rather than other factors.

There were several limitations to this study. Firstly, the relatively small sample size could be a contributing factor to the non-significant findings for most of the Bisphenols. Also, it has prevented us to split the sample in order to analyze influencing factors on overweight and obesity separately. Secondly, complication in the collection of saliva and nail samples resulted in fewer samples being submitted of these two matrices. In the case of nails, many of the children participating in the study tended to bite their nails, being a considerable drawback in their acquisition. In relation to saliva, some children refused to collect saliva because they were disgusted by it. However, the main strength of this study is that, to the best of our knowledge, this case–control study in Spanish children is the first to evaluate the possible association between BPA and 11 of its analogs, and overweight and obesity, analyzed in three distinct biological matrices (urine, nails and saliva). By not exclusively using a single biological sample, such as urine, to assess exposure to BPA and its analogs, we eliminate the risk of having one measurement that can bias our results via a misclassification of exposure due to, for example, the great variability of urine composition within a single day, or from 1 day to another.

The adverse health effects of BPA analogs should continue to be monitored, since BPA has already begun to be replaced by them, and human exposure to these alternatives will continue to increase. In addition, the limited evidence on the association between Bisphenols and overweight/obesity calls for further epidemiological and toxicological studies to assess whether human exposure to BPA substitutes increases the risk of overweight/obesity in children.

## 5. Conclusion

The current study is the first to report on the association between BPA and 11 of its analogs, and childhood overweight and obesity, analyzed in three distinct biological matrices (urine, nails, and saliva). The results suggest a positive association between BPF exposure in nails and a negative association between BPAF exposure in saliva. No associations of statistical significance were found between BPA and its analogs and overweight/obesity.

However, the contradictions between the associations of Bisphenols and adiposity in the previous studies require further epidemiological and toxicological studies, ideally of a longitudinal design and including Bisphenol measurements in different biological matrices that show long-term exposure, assuring that the concentration of Bisphenols in the samples does not fluctuate within the same day, or from day to day.

## Data availability statement

The original contributions presented in the study are included in the article/Supplementary material, further inquiries can be directed to the corresponding author.

## Ethics statement

This study was approved by the Ethics Committees of the University of Granada, the Provincial Biomedical Research of Granada (CEI), Spain (reference 1939-M1–22, Andalusian Biomedical Research Ethics Portal). The study was performed in accordance with the corresponding ethical standards. The studies were conducted in accordance with the local legislation and institutional requirements. Written informed consent for participation in this study was provided by the participants’ legal guardians/next of kin.

## Author contributions

YG-O and IM-R collected the data, performed the analyses, interpreted the data, and wrote the manuscript. VA provided the means for recruitment of the study population, collected the data, critically reviewed the manuscript, and gave final approval of the version to be published. CM collected the data, interpreted the data, revised the manuscript, critically reviewed the manuscript, and gave final approval of the version to be published. LR collected the data, supervised the writing, reviewed the manuscript, and gave final approval of the version to be published. AZ-G: conceptualized and designed the study, critically reviewed the manuscript, and gave final approval of the version to be published. RG-M provided project administration and funding acquisition. AR: conceptualized and designed the study, coordinated, supervised data collection, reviewed the manuscript critically, and gave final approval of the version to be published, as well provided project administration and funding acquisition. All authors contributed to the article and approved the submitted version.

## Funding

This study was carried out within the framework of the Plan Estatal de I + D + I 2013–2016, Proyecto cofinanciado PI20/01278, Proyecto cofinanciado FEDER-Consejería de Salud y Familias, Junta de Andalucía PE-0250-2019, Proyecto cofinanciado FEDER/Junta de Andalucía-Consejería de Transformación Económica, Industria, Conocimiento y Universidades/Proyecto P18-RT-4247, and Proyectos I + D + i del Programa Operativo FEDER 2020 CTS-252-UGR20.

## Conflict of interest

The authors declare that the research was conducted in the absence of any commercial or financial relationships that could be construed as a potential conflict of interest.

## Publisher’s note

All claims expressed in this article are solely those of the authors and do not necessarily represent those of their affiliated organizations, or those of the publisher, the editors and the reviewers. Any product that may be evaluated in this article, or claim that may be made by its manufacturer, is not guaranteed or endorsed by the publisher.
