# Supplementary material for: Levels of Bisphenol A and its analogs in nails, saliva, and urine of children: a case control study
Source: Front Nutr. 2023 Aug 14;10:1226820. doi: 10.3389/fnut.2023.1226820 (PMC10461051; doi:10.3389/fnut.2023.1226820)
Supplement: Supplementary file 1 [file Table_1.DOCX]

Supplementary Material

Exposure to bisphenol A and its analogues during childhood and its role in overweight and obesity.

Yolanda Gálvez-Ontiveros^1,2,3†^, Inmaculada Moscoso-Ruiz^2,3,4†^, Vega Almazán Fernández de Bobadilla^5^, Celia Monteagudo^1,2,3^, Rafael Giménez-Martínez^1,2^, Lourdes Rodrigo^2,3,6*^, Alberto Zafra-Gómez^2,3,4^, Ana Rivas^1,2,3†^

^1^Department of Nutrition and Food Science, University of Granada, 18071 Granada, Spain

^2^Instituto de Investigación Biosanitaria ibs.GRANADA, Granada, Spain.

^3^"José Mataix Verdú" Institute of Nutrition and Food Technology (INYTA), Biomedical Research Centre (CIBM), University of Granada, 18100 Granada, Spain.

^4^Department of Analytical Chemistry, University of Granada, 18071 Granada, Spain.

^5^Heath Center of Maracena, 18200 Granada, Spain.

^6^Department of Legal Medicine and Toxicology, University of Granada, 18071 Granada, Spain.

†These authors contributed equally to this work

*** Correspondence:** Dr. Lourdes Rodrigo: lourdesr@ugr.es

Number of pages: 2; Number of tables: 1

The supplementary materials contain supplementary a table for Material and Methods.

**Table S1.** Comparison between controls and cases included/not included subjects.

|  |  | Cases (n=94) | | | Controls (n=137) | | |
| --- | --- | --- | --- | --- | --- | --- | --- |
|  |  | Included (n=59) | Not included (n=35) | *p* | Included (n=101) | Not included (n=36) | *p* |
| Gender (%) | Male | 58.3 | 48.6 | 0.398^a^ | 49.5 | 50.0 | 0.999^a^ |
|  | Female | 41.7 | 51.4 |  | 50.5 | 50.0 |  |
| Age, categorized (%) | 6-10 years | 75.0 | 57.1 | 0.108^a^ | 79.2 | 88.6 | 0.312^a^ |
|  | >10-12 years | 25.0 | 42.9 |  | 20.8 | 11.4 |  |
| Weight, kg | Median | 53.30 | 51.20 | 0.758^b^ | 25.45 | 26.60 | 0.813^b^ |
|  | IQR | 21.90 | 25.70 |  | 12.58 | 10.13 |  |
| Height, cm | Mean | 140.37 | 141.67 | 0.682^c^ | 127.79 | 126.05 | 0.641^c^ |
|  | SD | 12.93 | 17.81 |  | 20.68 | 13.60 |  |
| Urinary creatinine, g L^-1^ | Median | 0.90 | - | - | 0.87 | - | - |
|  | IQR | 0.77 | - |  | 0.60 | - |  |
| IQR: interquartile range; SD: standard deviation; *p*-Values <0.05 are highlighted in bold; ^a^Chi-square test; ^b^U Mann-Whitney test; ^c^Student´s *t*-test | | | | | | | |
